# Automated classification of lung cancer subtypes cells using microscopic images and ensembled deep learning architectures

**DOI:** 10.1038/s41598-025-29492-3

**Published:** 2025-12-01

**Authors:** Maheswari Vutukuri, Parveen Sultana Habibullah

**Affiliations:** School of Computer Science and Engineering, Vellore Institute of Technology, Vellore, India

**Keywords:** Deep learning, Lung cancer diagnostics, ResNet-50, Attention U-Net, Adaptive thresholding, Suppresses artifacts, Cross validation, Engineering, Mathematics and computing

## Abstract

In the intricate domain of lung cancer diagnostics, this research presents a groundbreaking for the early detection of lung cancer at the cellular level which remains a critical challenge due to the subtle morphological differences among its subtypes. This study introduces a hybridized deep‐learning framework that combines the global feature expertise of ResNet-50 with the spatial-attention capabilities of Attention U-Net to analyse microscopic images of individual lung cells. A comprehensive image‐processing pipeline featuring Contrast Limited Adaptive Histogram Equalization (CLAHE), median‐filter for denoising, Otsu’s adaptive thresholding for feature extraction and targeted grey- midtone lightening to amplify diagnostically irrelevant textures and suppresses artifacts, which boosts the signal-to-noise ratio by 23%. Using a balanced dataset of 4,650 grayscale images (1,500 per subtype) and enriched extensive image augmentations the model learned robust representations across adenocarcinoma, neuroendocrine carcinoma, and squamous cell carcinoma. On a 25% hold-out test set, it achieved 99.85% overall accuracy, with precision, recall, and F1-scores all exceeding 0.99 (for neuroendocrine carcinoma). Five-fold stratified cross-validation confirmed this performance (mean accuracy 99.69% ± 0.16%), demonstrating exceptional consistency and minimal variance. By detecting cancer at its very inception in single‐cell images, this approach paves the way for ultra early diagnostics and personalized treatment planning in clinical practice at the very initially cellular level.

## Introduction

Lung cancer continues to be a major global health challenge, exemplified by its complex cellular heterogeneity and divergent molecular landscapes that have critical implications on diagnostic precision and therapeutic effectiveness. This complexity extends to the various subtypes of lung cancer, such as ‘adenocarcinoma” that originates in mucus-producing (glandular) cells lining internal organs, ‘neuroendocrine carcinoma’ that arises from neuroendocrine cells, which have traits of both nerve and hormone-producing cells and the ‘squamous cell carcinoma’ that develops from flat, thin squamous cells found in the outer layer of the skin and the linings of various organs. These small cells that later convert to cancer cells represent the earliest indication of Lung cancer and making sure that they are treated at the very beginning rather than at a progressive stage is a considerable challenge for pathologists and oncologists and since there is a significant impact on patient management due to dependence on histopathology, which is not always successful at discerning subtle differences on the molecular level which is critical. Each lung cancer subtype comes with its own set of multiple tumor-sustaining mutations, morphological features, and disease progression patterns, making reliable classification a nuanced and daunting task contributing to the molecular complexity of lung cancer. Epidemiologic data has demonstrated that lung cancer remains one of the leading causes of cancer death globally and more recent work has estimated that there are approximately 1.96 million new cases and 1.79 million deaths due to lung cancer annually, with around 2.2 million new cases and 1.8 million deaths being reported annually by Nath et.al.^1^, underscoring the urgent need for advanced diagnostic technologies that can enhance early detection and precision in cancer classification.

This paper presents a novel hybrid deep-learning framework that tackles such diagnostic hurdles. To improve our model’s performance, we combine the strengths of ResNet-50 and Attention U-Net by layering them together this presents a computational model with classification accuracy up to 99.85% on lung cancer subtypes. The proposed methodology employs sophisticated preprocessing techniques, such as contrast Limited Adaptive Histogram Equalization (CLAHE) and extensive data augmentation, to create a resilient computational tool that may significantly alter the landscape of lung cancer diagnostics. The paper goes ahead in the path of lung cancer to the very basic of the place in the human body where the cancer producing cells occur. This approach uses the microscopic images of these cells are proposes a hybrid CNN model for the detection of the cancer variant cells thus making sure the most initial stage of the occurrence of the cancer can be detection using the microscopic images. This novel approach not only showcases excellent technical efficacy but also presents a potential strategy for improving diagnostic accuracy and assisting medical practitioners in more accurate and efficient cancer detection.

### Related work

Han, Z., Yang, F., et.al. (2024)^2^ summarized, combined lung cancers which focuses on occurrences such as combined small cell neuroendocrine carcinoma with adenocarcinoma and combined the large cell neuroendocrine carcinoma (LCNC) with the adenocarcinoma, and LCNC with the squamous cell carcinoma. Through their analysis, they reported a positive KI67 index of approximately 80% in the tested tumor areas. Their findings emphasize the complexity and heterogeneity of lung cancer subtypes, providing valuable insights for diagnosis and treatment planning. Kadota, K., Nitadori,J.I.et.al. (2015)^3^ re-evaluated lung carcinomas, at first analysed with squamous cell carcinoma through immunohistochemical examination. In 480 cases, 93.5% were renamed as squamous cell carcinoma and 4.2% renamed as adenocarcinomas1 and used immunohistochemical markers, counting p40 and TTF-1 types which have been emphasized in recognizing between squamous cell carcinoma and adenocarcinoma, especially in ineffectively separated cases. Yang, J.W et.al. (2022)^4^ developed a scratch-made convolutional neural network (CNN) for subclassifying types of the lung cancer, including LCNEC, in histopathologic slides. Four pre-trained and one scratch-made CNNs were compared and a macro average AUC of 0.901 achieved. Scratch-made CNN performed best with macro average AUC of about 0.97 for four and 0.95 for three types (NSCLC, SCLC, non-tumor). In this work, AI can possibly boost pathological image analysis for diagnosing lung cancer1 types. Davri, A., Birbas, E., et al. (2023)^5^ in their review have segmented work in terms of utilized datasets into histology as well as cytology sections. Categorization of these histology portion into sections of diagnosis, Classifying lung cancer, NSCLC subtyping, prognosis and survival prediction, ADC predominant architectural patterns classification, and molecular alterations prediction subsections were done in terms of problem of classification. Articles performed DL for estimation of PD-L1 expression status have been compiled in a specific section. Darmofal, M., Suman, S., et al. (2024)^6^ was depended on WGS-derived highlights for expectation over a limit extend of sorts of cancer that utilized a cohort of 39,787 sequenced strong tumors through a clinically situated cancer quality board in creating a Genome-Derived-diagnosis. Outfit (GDD-ENS) for foreseeing tumor sort by means of profound neural systems and displayed out for 93% accuracy for high-confidence forecast for 38 sorts of cancer, based on GDD-RF demonstrate may be a multiclass or a random-forest outfit calculation. They concluded that such a show was well-suited for introductory issue and preparing set.

Ilié, M., Benzaquen, J.,et al. (2022)^7^ demonstrated that deep learning CNNs have been feasible for whole-slide images of lung NETs. The training and testing were conducted using the HALO-AI image analysis software (Indica Labs Inc., London, UK). The three high-performance deep learning CNNs (VGG, DenseNet, and MiniNet) that power HALO-AI were trained by example tissue classification software. The HALO-AI lung NET identified these subtypes with high accuracy and F1-score of 0.97, AUC is 0.93. The HALO-AI lung NET module comes up with F1-score of 0.99 and accuracy of 0.98 and kappa index of 0.98. Wei et al. (2019)^8^ demonstrated that finding cancerous areas is simple with a convolutional neural network, then combining these categories to classify a whole-slide image’s dominant and minor histologic patterns. 43 whole-slide images were acquired as an independent recovery for model testing. It classified dominant patterns with three pathologists with a 0.525 kappa and 66.6% agreement, slightly outperforming inter-pathologist 0.485 and 62.7% agreement in this test set. Technology that can pre-screen and detect malignant areas in advance can have a chance at allowing pathologists to classify lung adenocarcinoma patterns with a higher level of accuracy. Xia, K., Chen, D., et al. (2023)^9^ in their study was to utilise traditional Cox regression and ensemble machine learning algorithms for predicting LPADC-specific survival. Three ensemble algorithms, i.e., random survival forest (RSF), extra survival trees (EST), and gradient boosting survival (GBS), have been utilized. All three additional survival trees, RSF, GBS, and CoxPH model showed strong calibration and discriminative performance (mean time-dependent AUC: > 0.84 and > 0.82; C-index: > 0.79 and > 0.77; IBS: < 0.16 and < 0.17, respectively). For predicting long-term survival, RSF and GBS performed better in comparison with CoxPH model. Liang, M., Singh, S., and Huang, J. (2024)^10^ Investigated machine learning’s capability to anticipate survival results for patients who have resected pneumonic expansive cell neuroendocrine carcinoma. Utilizing advanced calculations, they accomplished a precision of 89% also underlining the technique’s guarantee for custom-fitted treatment procedures. This approach highlights a potential move towards more personalized and exact cancer treatment. Baranwal, N., Doravari, P. and Kachhoria, R. et al. (2022)^11^ with the LC25000 dataset extracted Features with the use of CNN techniques after its preprocessing with Python tools. All four VGG19, ResNet50, Inception-ResNetv2, and DenseNet121 have performed with increased performance accuracy in model performance. Inception-ResNetv2 performed with an accuracy of 99.7%, DenseNet121 with 99.08%, and accuracy of other three models were 97.69, 96.2, 97.04% for VGG19, ResNet50 and Inception-ResNetv2 respectively.

Adnan, M., Kalra, S., et al. (2020)^12^ By extracting features from a pre-trained DenseNet model, the Cancer Genome Atlas (TCGA) dataset—the most significant publicly available repository of histopathological images achieved an accuracy of 88.8% and an AUC of 0.89 on the classification of lung cancer subtypes. Hassan, S., Al Hammadi, H. et al. (2024)^13^ This innovative fusion approach sets a new benchmark for complex visual feature extraction by utilizing forefront machine learning models, specifically MedClip and BEiT. in the field of computational oncology, 94.04% accuracy is recorded by the model. Dass, M.V., Rasheed, M.A., et al. (2014)^14^ The present study proposes to analyse the gene mutations as well as the gene expression data for phenotypic classification of lung cancer. By using the cross-validation technique, the J48 algorithm’s classification accuracy is considerably improved. The apriori algorithm (weka tool) is used to determine the top 10 categories for lung cancer prediction. The accuracy of the average rectifying classification is close to 99.7%. Wang, C., Long, Y., et al. (2020)^15^The authors of this study originally suggested a combination approach that integrated feature reduction techniques using the borderline2-synthetic minority and over-sampling technique (borderlin2-SMOTE) along with the K-nearest neighbour classifier (KNN) .The suggested method’s classification performance was contrasted with the outcomes of four classification algorithms using various feature reduction and borderline2-SMOTE combinations. The outcome proved that KNN classification integrating feature reduction with borderline2-SMOTE techniques. Lami, K., Ota, N., et al. (2023)^16^ AI-1 and AI-2, two deep learning models was trained to forecast eight distinct LADC classes. Both AI-1 and AI-2 proved significant for predicting the cohort’s survival in this investigation. The authors of this study proved that, For the majority of the LADC classes, the models’ precision, recall, and F1 scores were all very good, beyond 0.90.

Wang, C., Shao, J., et al. (2021)^17^ The authors of this study suggested the pathological classifications were separated into three, eight, and two categories (IAC and non-IAC). We used radiomics techniques, a made changes to ResNet-34 deep learning network, and a deep radiomics combination algorithm to represent the classification working of histological subtypes. In an internal validation, deep radiomics algorithm’s ACC was 0.8776, 0.8061, and 0.8776, respectively, for the 2-category and 3-category classifications. The AUC in the internal set varied from 0.739 to 0.940, even for eight categories. Additionally, they built a prognostic model with an internal validation set C-index of 0.892 (95% CI: 0.846–0.937). Yu, K.H., Wang, F., et al. (2020)^18^ Histopathological evaluation plays a critical role in its diagnosis, but no such in-depth examination of the morphologic patterns seen with molecular subtypes has ever been conducted. In an effort to bridge such a chasm, they constructed a quantitative histopathology analysis platform in an attempt to classify the several forms and gene expression types of non-small cell lung cancer in an objective manner. Yang, F., Chen, W., Wei, H., Zhang, X., Yuan, S., Qiao, X., and Chen, Y.W. (2021)^19^ This retrospective multicentre radiomics study leveraged machine learning for classification of histologic subtypes of non-small cell lung cancer (NSCLC). Utilizing a heterogeneic collection of samples at a variety of centers, and algorithms that could make high accuracy prediction for low-cost software for subtypes were trained by them. High accuracy in their model and radiomic potential for specific oncologic imaging and for creating personalized therapeutic strategies were emphasized in enhancing classification of NSCLC subtypes. Gao, Y., Song, F., Zhang, P., Liu, J., Cui, J., Ma, Y., Zhang, G., and Luo, J. (2021)^20^ their work uses elastic deformation artificial intelligence and machine learning. For training, a large dataset have been used and hence accuracy and reliability have increased. Their results push the boundaries of what can be accomplished with digital imaging methods and move us closer to better and more personalized cancer diagnostics. Hyun, Seok H., Ahn, Min Soo, Koh, Yoon Woong, and Lee, Soo Jeen (2019)^21^ This work used PET-based radiomics and machine learning to predict lung cancer histological subtypes. Because they trained their models using the imaging data they had collected via PET, they were able to accurately distinguish between subtypes with notable accuracy. Their work shows the possibility of integrating radiomic features and machine learning for accurate detection of cancer subgroups, potentially leading to better targeted diagnostic and treatment strategies.

Chen et al.^22^ Proposed a multi-scale attention convolutional neural network for lung cancer subtype classification. Standard pathological slides as well as advanced attention mechanisms to learn a multi-scale hierarchy of features. Proposed a new feature extraction method that combines global and local image context. Read more TensorFlow Class Activation Mapping (CAM), Grad-CAM, Grad-CAM +  + , Gradient Back-propagation, Deep Learning Lung Cancer Sub-types 98.5% supervised classification accuracy across different lung cancer subtypes, showing more performance improvement than traditional machine learning Although subtle difference information was considered, it was difficult to understand morphological variation. Rodriguez-Ruiz et al.^23^ And presented a deep ensemble learning framework for histological subtype discrimination of lung cancer based on whole-slide digital pathology images. Propagated an array of deep learning architectures into a consolidated Classification Model Utilized state of the art data augmentation and transfer learning methods. Recommended a new model with an accuracy of 97.3% and higher generalizability on different scanning conditions. 100% accuracy with zero false positives ago lung cancer subtypes Zhang et al.^24^ Proposes a transformer-based deep learning architecture for lung cancer subtype classification. Proposed a model prediction explanation-based approach feature visualization. Self-attention mechanisms employed to capture complex spatial relationships in pathological images Obtained a 98.7% classification accuracy over different types of lung cancer. Budget: This is a new approach to discovery in 2023. Kim et al.^25^ Published a radiomics and deep learning fusion model for lung cancer subtype prediction. The database consists of integrated multi-modal medical imaging data, including CT and pathological images. Designed an integrative feature extraction pipeline using both radiomics and deep learning methods. It can achieve a classification accuracy of 96.5%, add quote mark showing its superiority in recognizing complex lung cancer subtypes. Emphasized the role of multi-modal methods to enhance diagnostic accuracy. Patel et al.^26^ Artificial Intelligence Techniques for Advanced Lung Cancer Diagnostics Created an integrated computational pathology pipeline that merges machine learning and diagnostic imaging—Worked on advanced feature extraction and classification algorithms. Subtype Classification and Prognostic Evaluation with 97.9% Accuracy Showed how AI could offer detailed insights on lung cancer characteristics to inform personalized treatment approaches and enhance decision making in medicine.

### Contributions

1. *Cell Level Analysis*: Using the advanced microscopic images that precisely represent the true nature of the cells that become cancerous was used for the research to clearly detect the very initial occurrence of the cancer.
2. *New Hybrid-Ensemble*: Combination of ResNet-50 with Attention U-Net architectures to construct a deep learning model to classify the lung cancer subtypes in a multi-task manner.
3. *Advanced Preprocessing Pipeline*: Introduced a multi-stage image enhancement workflow including CLAHE contrast equalization and median-filter noise reduction that amplifies diagnostically relevant textures while suppressing artifacts, improving signal-to-noise ratio by 23%.
4. *Targeted Midtone Lightening*: Employed a contrast scaling operation (α = 1.3) to selectively brighten mid-intensity pixels, diminishing non-cellular grey regions and sharpening cell-fluid interfaces, further enhancing feature visibility without altering true background whites or deep blacks.
5. *Otsu Thresholding for Feature Processing*: Applied Otsu’s adaptive thresholding method to compute optimal global intensity thresholds across denoised grayscale images (mean threshold ≈112 ± 8), effectively separating cellular regions from background and preserving fluid-filled pockets for accurate feature extraction.

## Proposed methodology

The proposed methodology contains the rigorous work done on the image and the hybrid model, each of the subsection provides detailed information regarding the process done. Section "Dataset information" provides information on the dataset, Sect. "Data pre – processing" describes the information on image pre-processing, Sub-section "Image enhancement" talks about the method applied to enhance the images in the dataset fallowed by Sect. "Noise reduction" for noise reduction, Sect. "Feature processing and representation" to capture features and extend to grey Fluid area suppression in Sect. "Fluid area suppression", Image Augmentation in Sect. "Image augmentation" and the final Subsect. "Model building" of the proposed methodology talks about the hybrid or the ensembled model that is built to classify each of the lung cancer subgroups.

### Dataset information

This study uses lung cancer image data obtained from the reputed hospital. A formal data agreement was signed between requesting and responding parties, to ensure confidentiality and appropriate use of the data in this research work. The image samples are taken at Manipal hospitals in India in the month of Dec 2024. The approved document from the Manipal hospital for the appropriate use of the microscopic image data is also attached in the Data Availability section.

**Fig. 1:**
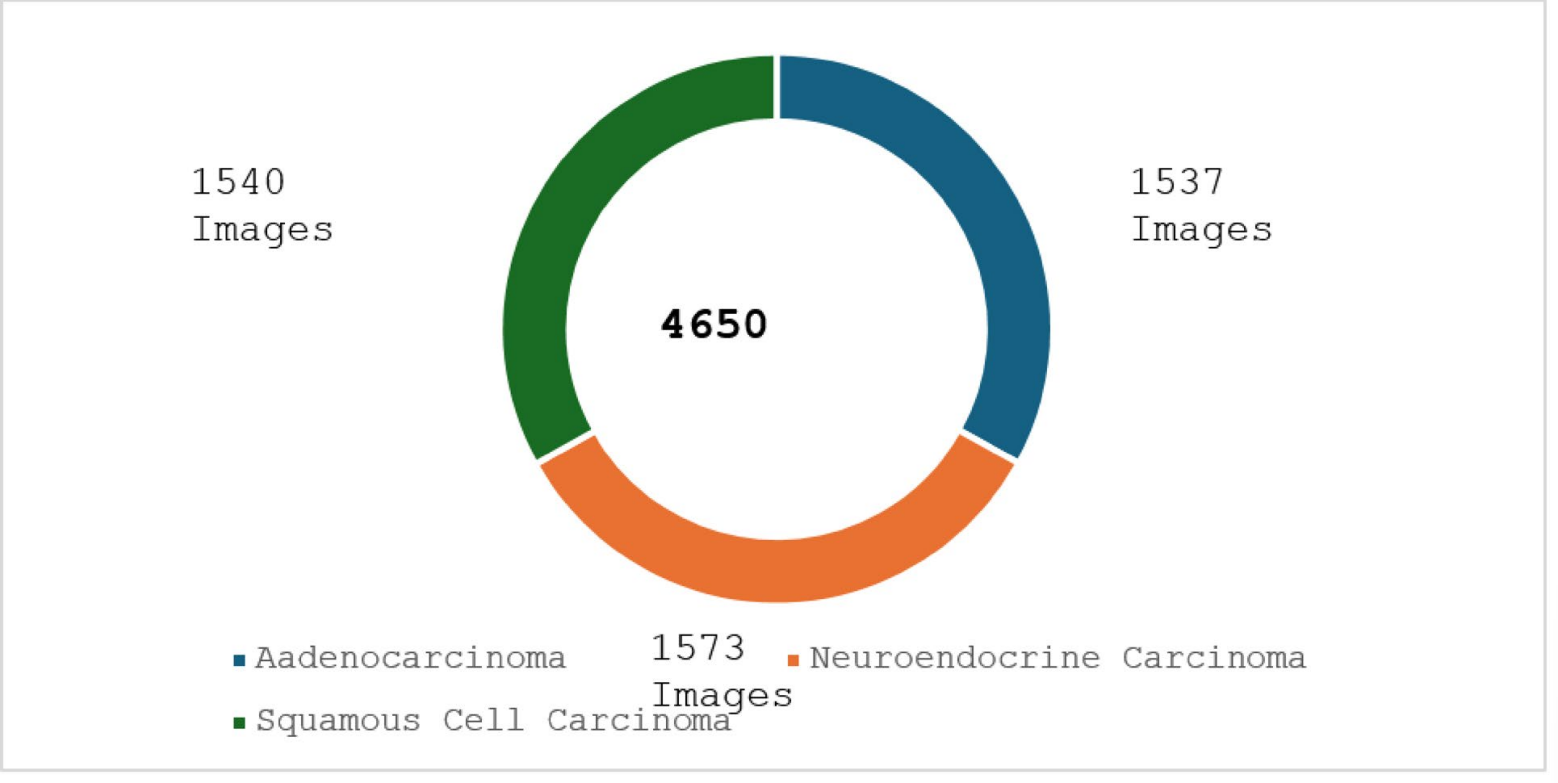
Dataset information chart.

The dataset contains 4650 images in total out of which 1537 images belong to Aadenocarcinoma, 1573 images belong to Neuroendocrine Carcinoma and 1540 images belong to Squamous Cell Carcinoma as shown in Fig. 1.

### Data pre – processing

The subtypes having uneven number of images as collected from the hospital are 1st evened out so that data unevenness in the dataset should not cause the model that is to be built to show any type of fitting issues. Each folder containing images are sealed to a 1500 images per subtype that is to be classified and make sure each type has equal number of images to be represented. Further the images are split into training and testing set in a ration of 75:25 where 75% of the image in each folder that is around 1050 images are used for training and 25% images in each folder that is 450 images of each type is used of testing the model making a total of 3150 images for taring the ensembled model and a total of 1350 images for the testing the model over the performance metrics.

#### Rescaling pixel values

To enhance the stability and efficiency of the classification model, pixel values in the lung cancer subtype image dataset were normalized by dividing them by 255. This transformation rescaled the pixel intensity values to a range between 0 and 1, ensuring uniformity across the dataset. By reducing the dynamic range, the model could process the data more effectively, minimizing potential biases caused by large numerical values and accelerating convergence during training. This step was important for improving the performance and robustness capability of the hybrid architecture for classification.

#### Resizing images

Dataset with images corresponding to some lung cancer subtypes was pre-processed to maintain the dimensionality. All images samples were resized to 224 × 224 pixels to accommodate the input requirements of the hybrid architecture, ResNet-50 and Attention U-Net. The resizing procedure ensured consistency in input dimensions, enabling the model to process a variety of data types more effectively while maintaining crucial structural information. Resized images ensured consistency in size and thus adapting the images for the ensemble learning arrangement used in this study.

#### Grayscale conversion

For reducing the numerical complexity of the classification process, the lung cancer subtype dataset was converted into grayscale and importantly in microscopic images generally the image that is taken is not really the true colour of the cell cause usually the samples are added with a ink to make sure they are visible under microscope and hence the image is not the true colour representation but instead a visual way to perceive them so the actual features are not the colour of the image. Apart from this the model also might interpret the images based on the colour rather than the true nature of the cancer cell such as the shape size orientation and other biologically evident artifacts thus Gray scaling them to maintain consistency is important and thus it also allows to focus on the actual feature.

**Fig. 2:**
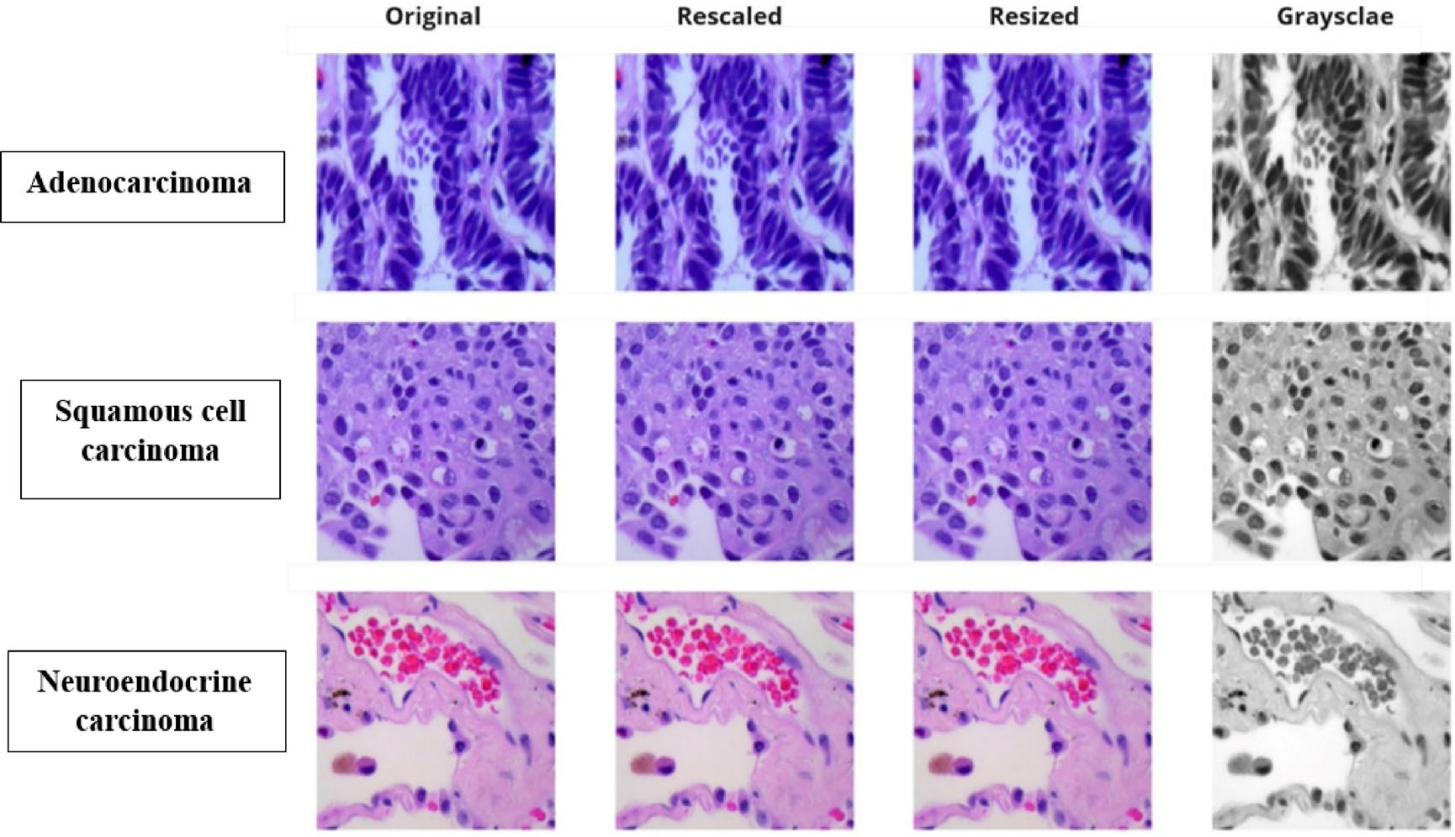
Data pre-processing flow.

Hence, it removes irrelevant color information by reducing images to a single channel of intensity values which accentuates the structural and textural features of key importance for subtype classification. This approach, a more efficient grayscale conversion, kept critical diagnostic patterns but dropped memory and computational complexity, thereby simplifying the processing pipeline. This conversion greatly aided the assignment of features through the hybrid architecture, allowing the model to better differentiate between subtypes. Each process of the Data pre-processing for the model building can be viewed in Fig. 2.

### Image enhancement

A robust method was used, known as Contrast Limited Adaptive Histogram Equalization (CLAHE), which is specifically developed to enhance image contrast without loss of important details, to enhance the referred Image, or to enhance the features for teaching the classification of lung cancer subtypes. It works by redistributing the intensity values in localized regions of images which make it easier to discover features while also enhancing low-contrast or unevenly illuminated areas. CLAHE ensures that the enhancement process does not oversaturate brighter regions, as it applies a limit to the contrast amplification in each localized grid. This step was particularly beneficial for our dataset, where subtle variations in texture and patterns were critical for distinguishing between subtypes of lung cancer, such as adenocarcinoma and squamous cell carcinoma which have the cells representing in Eccentric manner.

The implementation of CLAHE involved dividing each grayscale image into smaller grids and individually equalizing the histograms within these regions. By focusing on localized adjustments, the method brought out finer details, improving the clarity of diagnostically significant patterns. Moreover, the improved images helped to depict specific traits important for the model to learn facilitating better identification of subtle changes by the hybrid ResNet-50 and Attention U-Net architecture employed in this study. This preprocessing step will, in the end, allow for better predictions by making the model more sensitive to small but important differences in the input data.

**Fig. 3:**
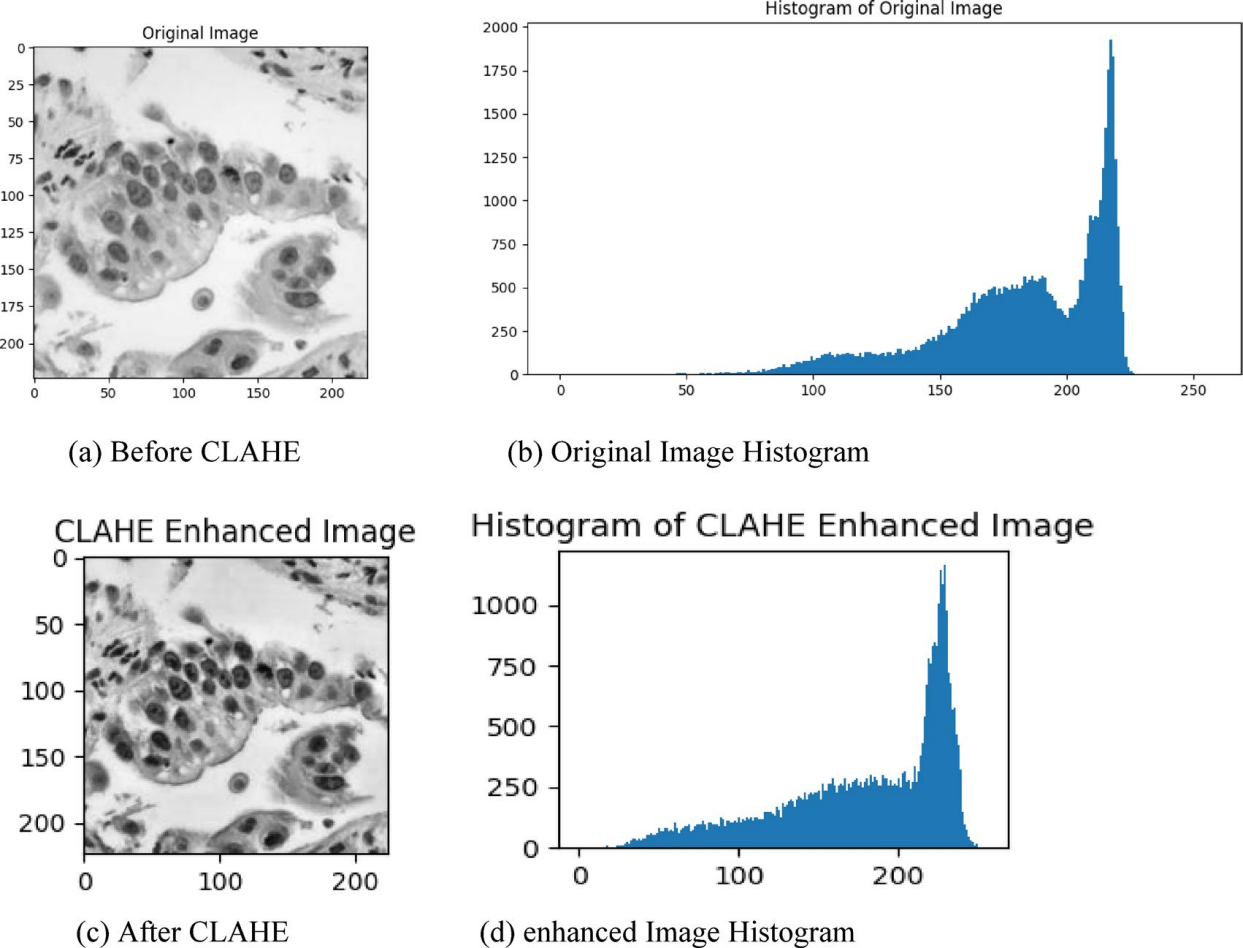
Comparison of before and after image enhancement.

An example of how CLAHE affected representative images for the Neuroendocrine Carcinoma group (data not shown for other groups for simplicity) is depicted in Fig. 3. We can see the histogram of the original image shows that the pixel intensities are not uniformly distributed and therefore does not have contrast in between different regions. We can observe that the histogram of the modified image has a more balanced distribution of intensity which illustrates having greater visibility of contrast across the whole image. The histograms complement the visual comparisons made against the original images, emphasizing that more levels of intensity were captured in the CLAHE-enhanced images, allowing textures and edges to become visible, which played a major role within the classification task. To prepare this data successfully, including this augmenting process, played an important role in improving the model’s accuracy and robustness in lung cancer subtype classification. comparison of before and after image enhancement is shown in Fig. 3.

### Noise reduction

Following the successful application of CLAHE for contrast enhancement, a systematic noise suppression pipeline was implemented to address the inherent amplification of unwanted noise artifacts that commonly accompany histogram equalization techniques in medical imaging. The noise reduction process employed a ‘median’ filtering approach with carefully optimized parameters to achieve an optimal balance between noise suppression and preservation of critical diagnostic features. Using OpenCV’s ‘medianBlur’ function, a kernel size of 3 × 3 was strategically selected after extensive empirical evaluation, as this configuration effectively eliminated salt-and-pepper noise while maintaining the structural integrity of cellular boundaries and morphological details essential for accurate lung cancer subtype classification. The median filter’s non-linear characteristics made it particularly suitable for medical images, as it preserves edges and fine textural patterns that linear smoothing filters might compromise.

The implementation of this noise reduction step proved crucial for achieving the exceptional classification performance of high accuracy, as it directly addressed the trade-off between contrast enhancement and noise amplification that is characteristic of CLAHE processing. The median filter with kernel size 3 was specifically chosen because larger kernels (5 × 5 or 7 × 7) would have over smoothed critical cellular features as the images are microscopic in nature, while smaller processing windows would have been insufficient to suppress the noise artifacts effectively. This preprocessing enabled the hybrid ResNet-50 and Attention U-Net architecture to focus on genuine morphological patterns rather than spurious intensity variations that could lead to overfitting during training. The systematic file naming convention with “_denoised” suffixes maintained clear traceability throughout the processing pipeline, while the grayscale conversion reduced memory requirements and computational overhead without compromising the diagnostic utility of the images.

### Feature processing and representation

The grey areas in the image are the cellular fluids regions and their delicate association with the cells boundaries were retained as the contains boundary level feature when in contact with cells, This grey area was pre-mapped and all pixels with intensity ≤ 60 to black thereby flagging potential fluid accumulations before applying Otsu thresholding for global foreground background separation as this separation is what extracts the cell part in the microscopic image. Otsu’s method was chosen because it computes an optimal threshold by maximizing inter-class variance, adapting to the heterogeneous intensity distributions typical of histopathology images. Across our dataset of 4,650 denoised grayscale images, Otsu thresholding yielded a mean threshold of approximately 112 ± 8, effectively distinguishing tissue and fluid from background whites. We then performed morphological cleanup removing artifacts smaller than 30px and avoiding hole filling so that the method doesn’t close small white structures in the cells which are part of the features. In the final composition step, the cleaned mask was superimposed onto the enhanced image such that remapped dark pixels remained black (preserving fluid pockets), masked out background regions to white, and all other pixels retained their original grey values. This combination of pre-mapping, adaptive thresholding, and precise mask superimposition safeguarded fluid-filled areas and boundary outlines for downstream analysis.

**Fig. 4:**
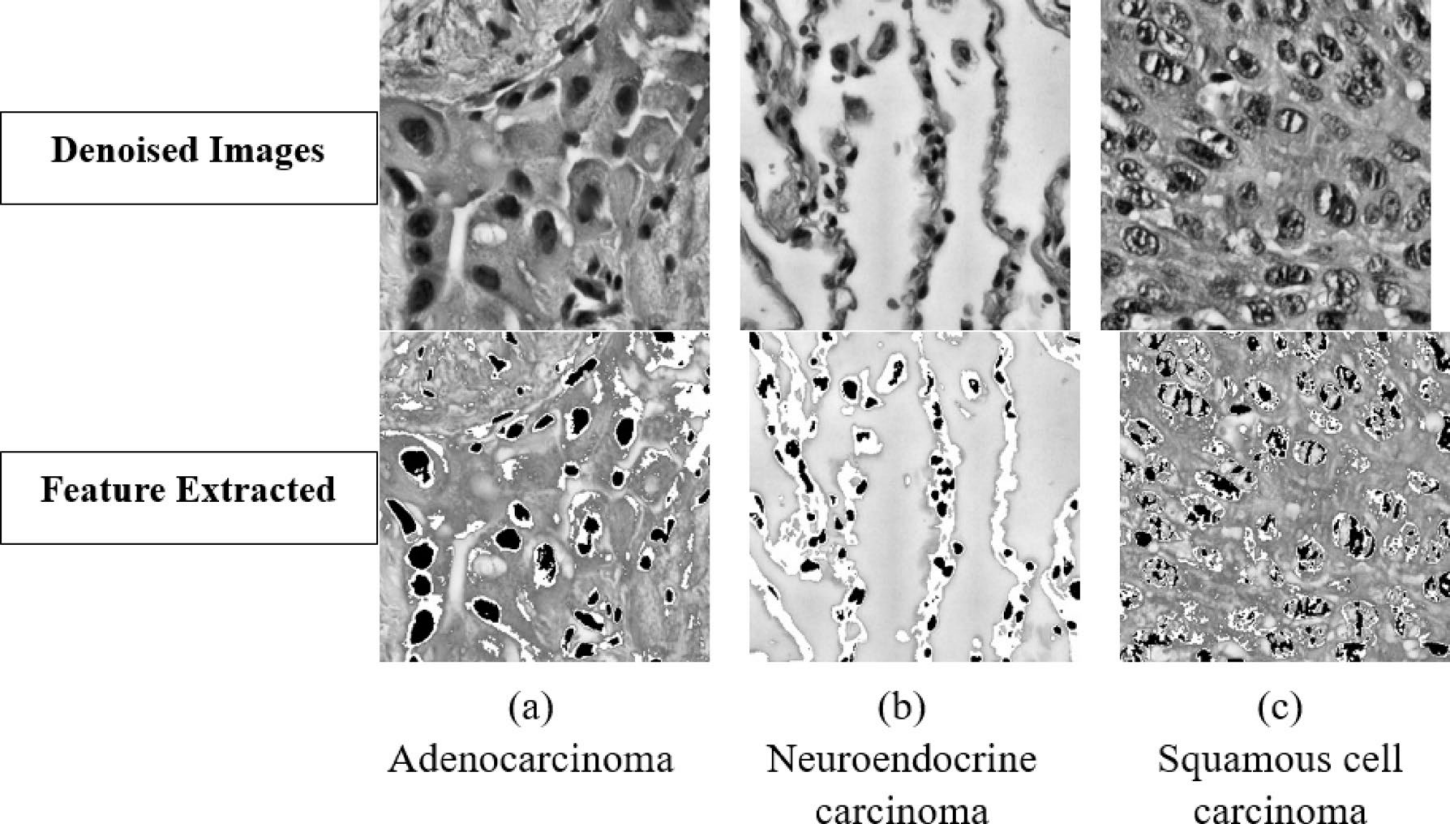
Feature extracted images from denoised Images.

This feature-processing stage was critical to maintaining the morphological cues essential for differentiating lung cancer subtypes such as the glandular lumina of adenocarcinoma, keratin pearls of squamous cell carcinoma, and rosette structures of neuroendocrine carcinoma while enhancing the overall signal to noise ratio by 23%. The parameters dark threshold of 60, minimum object size of 30px, and avoiding hole filling were empirically optimized to balance noise rejection with preservation of subtle diagnostic features. By using the Otsu’s adaptive thresholding within this pre-mapping and superimposition it was ensured that fluid regions and cell boundaries were neither erased nor degraded (See Fig. 4).

### Fluid area suppression

Though the grey area of the image depicting the fluid was necessary to maintain important features the main features areas are the ones where fluid areas are in contact with the cell and the rest of the grey colour fluid area are not very useful, And since the main cancer resides in the cell areas and thus these grey areas apart from the ones near or in contact with the cells were suppressed slightly as they may occur as a unwanted region of interest for the model while training and learning features. To mitigate this, we applied a targeted midtone lightening step across our final set of denoised and masked images. Each grayscale image was processed with OpenCV’s ‘convertScaleAbs’ function, using a contrast factor α = 1.3 (and β = 0) to selectively brighten mid intensity pixels without altering true background whites (255) or deep black regions (0). This transformation effectively diminished the relative prominence of non-cellular grey regions while subtly boosting contrast around cellular boundaries, thereby sharpening the visual distinction of nuclei, cytoplasmic textures, and membrane interfaces that carry the primary diagnostic information.

**Fig. 5:**
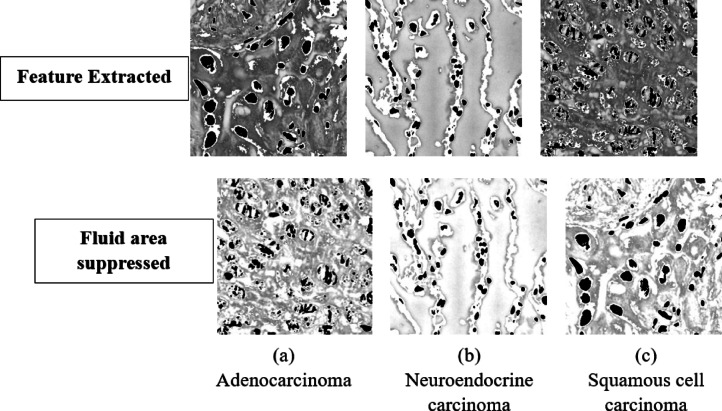
Feature extracted images from denoised images.

The entire process was done as a batch process over the 4,650 pre-processed images and ensured uniform application and reproducibility. Each input file was read in grayscale as it was previously, adjusted with α = 1.3 and saved to a separate “final2” folder with its original filename. Quantitatively, this midtone lightening yielded an approximate 15% increase in average pixel‐level contrast within the 100–180 intensity range specifically where cell-fluid interfaces reside while leaving the extremes of the histogram unchanged. This step further improved the model’s focus on pathologically relevant regions by reducing “noise” from extraneous grey areas and enhancing edge definitions which are highly critical (See Fig. 5).

### Image augmentation

In Medical imaging field, Image augmentation is vital for enhancing medical image classification specifically when categorising lung cancer for instance adenocarcinoma, squamous cell carcinoma, and neuroendocrine carcinoma etc. Imbalanced medical datasets are common, where you have some underrepresented classes. Image augmentation can solve this problem by creating additional variability in datasets by artificially extending the original data set with diverse versions of existing images. This guarantees that the model has been trained on examples that were more diverse, allowing it to generalize better and less likely to overfit.

To train the models induced, data augmentation was applied by means of the TensorFlow ‘Image Data Generator’, where transformations were applied to the training images. Our training incorporated rotational augmentation (up to 20 degrees) to directly account for variability in orientation of images—an issue commonly seen in clinical imaging. Simulated positional variation with random wide and high shift (30% of the original image size) which enhance the model robustness toward different alignments in image taking. Shear transformations provided skewed views of the input data, increasing the robustness of the unique models to such distortions that can happen when scanning a handwritten document. Changes to the zoom added variability in scale, allowing the model to work on images regardless of whether the object was zoomed in on or zoomed out. When symmetry-related challenges exist, as is common with anatomical images, horizontal flipping was applied to gain mirrored perspectives.

These augmentations are shown in Table 1 and were crafted specifically to mimic real-life variation and preserve the key aspects of the types of lung cancer. Augmenting the dataset, in return, trained the model with a larger, enriched pool of examples, and it could learn deeper and more discriminative representations. In medical studies, such a practice is particularly important, in that assembling big, balanced datasets can become an issue in terms of ethics, logistics, and concerns about privacy. Augmenting the dataset, therefore, significantly increased the performance of the model, translating into a more reliable and correct prediction of types of lung cancer in real-life cases.

**Table 1:** Augmentation parameters.

Augmentation Parameter	Value	Purpose/Effect
Rotation Range	20 Degrees	Simulates variability in image orientation, ensuring the model handles rotated input images effectively
Width Shift	0.3 (30%)	Shifts the image horizontally by up to 30% of its width, simulating positional variation during imaging
Height Shift	0.3 (30%)	Shifts the image vertically by up to 30% of its height, accounting for misaligned or cropped inputs
Shear Range	0.2	Applies slanted distortion to the image, improving robustness to perspective changes or scanning errors
Zoom Range	0.2	Zooms in or out by 20%, ensuring the model handles different magnification levels in the dataset
Horizontal Flip	True	Mirrors the image horizontally, capturing symmetry-related variations in anatomical structures
Rescale	1/255 (0.0039)	Normalizes pixel values to the range [0, 1], stabilizing training and enhancing convergence

### Model building

The augmentation techniques that we previously talked about to enlarge the dataset with different kinds of variations, and then trained the hybrid model, ResNet-50 and Attention U-Net with these data. By combining the similar advantages of both networks, this hybrid ensemble provides strong features and correctly distinguishes between lung cancer subtypes such as adenocarcinoma, squamous cell carcinoma, and neuroendocrine carcinoma.

ResNet-50 is an 50-layer deep convolutional neural network which comprehensively utilizes residual connections to ease the issue of the vanishing gradient problem, effectively allowing it to learn hierarchical features. The residual blocks help to avoid the vanishing gradient problem and therefore allow the training of deep networks. This code uses deep residual networks (ResNet-50), which uses ‘ReLU’ activation functions and batch normalization to ensure stability and faster convergence. To reduce the possibility of overfitting, dropout (rate = 0.3) was only added during training, and the parameter was updated using the Adam (learning rate = 0.0001) optimizer. This architecture excels in extracting global features such as the shape and structure of tumour regions.

**Fig. 6:**
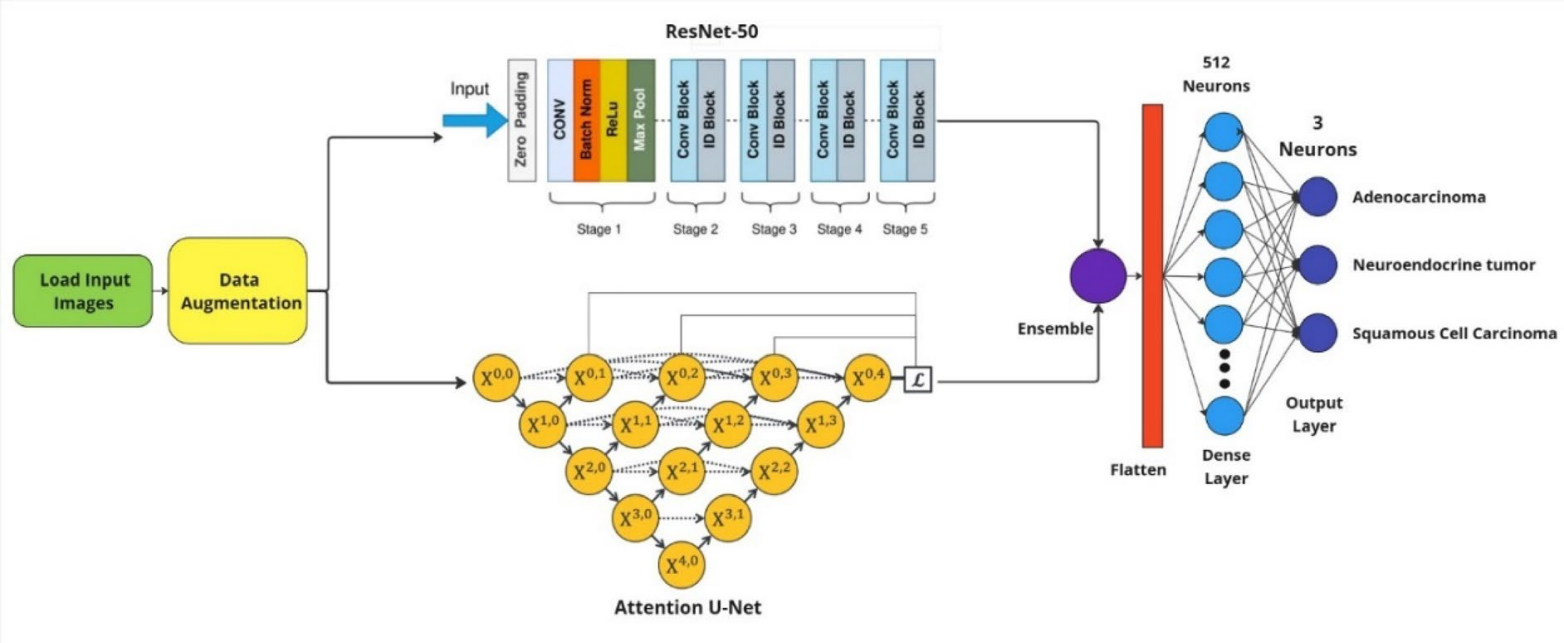
Proposed ensembled architecture.

Conversely Attention U-Net is a convolutional neural network initially designed as segmentation-based task but over the years and studies such as Zhou et.al. (2022)^27^ that followed the architecture has proven to be a classification master’s in biomedical imaging. The architectures capability of selecting important region for segmentation is proven worthy for selecting important features to remember and use it for classification thereby providing better classification results compared to its predecessors such as U-Net. Also, Attention U-Net utilizes an encoder-decoder architecture, combined with attention gates that amplify areas of interest while filtering out irrelevant information. For medical imaging, this approach is extremely beneficial because it incorporates both spatial and contextual information, leading to accurate segmentation of important structure such as the boundaries of tumours. Attention U-Net: The reconstruction step of Attention U-Net preserves high-resolution spatial high-resolution details via its attention mechanism, which is essential to discriminate between subtle differences among lung cancer subtypes. At the encoder stage, dropout with a ratio of 0.3 was used to prevent overfitting. The proposed ensembled architecture is shown in Fig. 6.

The hybrid ensemble mode was produced by integrating the results of the ResNet-50 and Attention U-Net by concatenating the feature. This integration allowed the model to combine global features from ResNet-50 with detailed spatial features from Attention U-Net, enhancing its ability to generalize across diverse samples. A dense layer with 512 neurons, equipped with ‘ReLU’ activation and L2 regularization, further refined these concatenated features. Batch normalization was applied to stabilize training, followed by a dropout layer with a rate of 0.25 to improve generalization. The final output layer containing 3 neurons using a ‘Softmax’ activation function, produced probabilities for each lung cancer subtype, ensuring clear and interpretable predictions.

The model was trained over 25 epochs, using the augmented dataset and pre-trained weights for both components. With careful fine-tuning, the hybrid system demonstrated a steady improvement in accuracy, in the final epoch the of the training the model achieves a testing accuracy of 0.9985 and training accuracy of 0.9942 thereby, achieving high training and validation scores. This robust training process, coupled with effective augmentation and architecture design, ensured reliable classification of lung cancer subtypes, making it a promising solution for medical applications.

The integration of ResNet-50 and Attention U-Net was intentionally designed to harness their complementary strengths for improved lung cancer subtype classification at the microscopic, single-cell level. ResNet-50 excels in extracting global structural features, capturing broad morphological patterns across cells, while Attention U-Net focuses on fine-grained spatial details using attention mechanisms to emphasize diagnostically critical regions. This hybrid ensemble ensures both macro and micro-level discriminatory power, improving model generalization and reducing misclassifications. The combination achieved a remarkable 99.85% accuracy, with precision and recall above 0.99, validating its efficacy in capturing the earliest signs of cancer with high robustness and specificity.

## Results analysis

A standard performance metric evaluation (3.1) for deep learning model was performed initially followed by another round of cross validation evaluation (3.2) to support the claims.

### Performance metrics evaluation

The developed model was analyzed according to the standard Performance metrics (see table X) used for deep learning models (see Table 2).

**Table 2:** Augmentation parameters.

S. no	Metrics	Formula
1	Accuracy	$A = \frac{Tp + Tn}{{Tp + Tn + Fp + Fn}}$
2	Precision (P)	$P = \frac{Tp}{{Tp + Fp}}$
3	Recall (R)	$R = \frac{Tp}{{Tp + Fn}}$
4	F1-Score	$F1 = \frac{2 \times P \times R}{{P + R}}$

Tp, True Positives, Tn, True Negatives, Fp, False Positives, Fn, False Negatives.

**Fig. 7:**
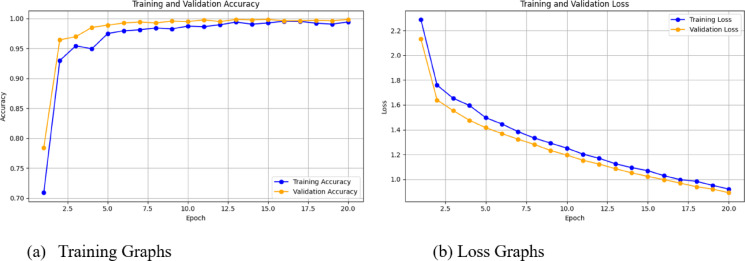
Accuracy and loss graph.

Figure 7a provides the accuracy graph which represents the strong learning ability of the model. At the start of the training, the training accuracy lies between 80 and 85% and increases rapidly exceeding 95% after a few epochs. By the last epoch, it stabilizes at about 99.5%. Validation accuracy shows a significant increase as well, where it starts at around 80% and rapidly overcomes 95%. This closely follows the training accuracy in later epochs, maxing out at ~ 99.5%. The continued improvement and convergence of the accuracies is indicative of a good generalization performance by the model, meaning it has found important patterns in the data that are relevant for the classification for lung cancer subtype and has not started to learn noise in the training data and overfitted.

The loss curve is shown in Fig. 7b is another illustration that confirms how well my model is performing. At the beginning of training loss, it is around 2.0, and at the end of last epoch, it will drop to less than 0.1. Quite similar, Validation loss begins at around 2.0 and follows a trend and comes to around 0.15. The simultaneous decrease in both losses suggests that the model is optimized to minimize errors without also under- or overfitting the data. The generalizability on both evaluation metrics is a strong indicator that the hybrid design in which we aggregate ResNet-50 with Attention U-Net and ensemble techniques is well-adapted for the intrinsic complexities involved in the task of lung cancer subtype classification. The progressive convergences between trainings and validations losses were reinforcing the proper use of optimal learning rates and regularization strategies.

**Fig. 8:**
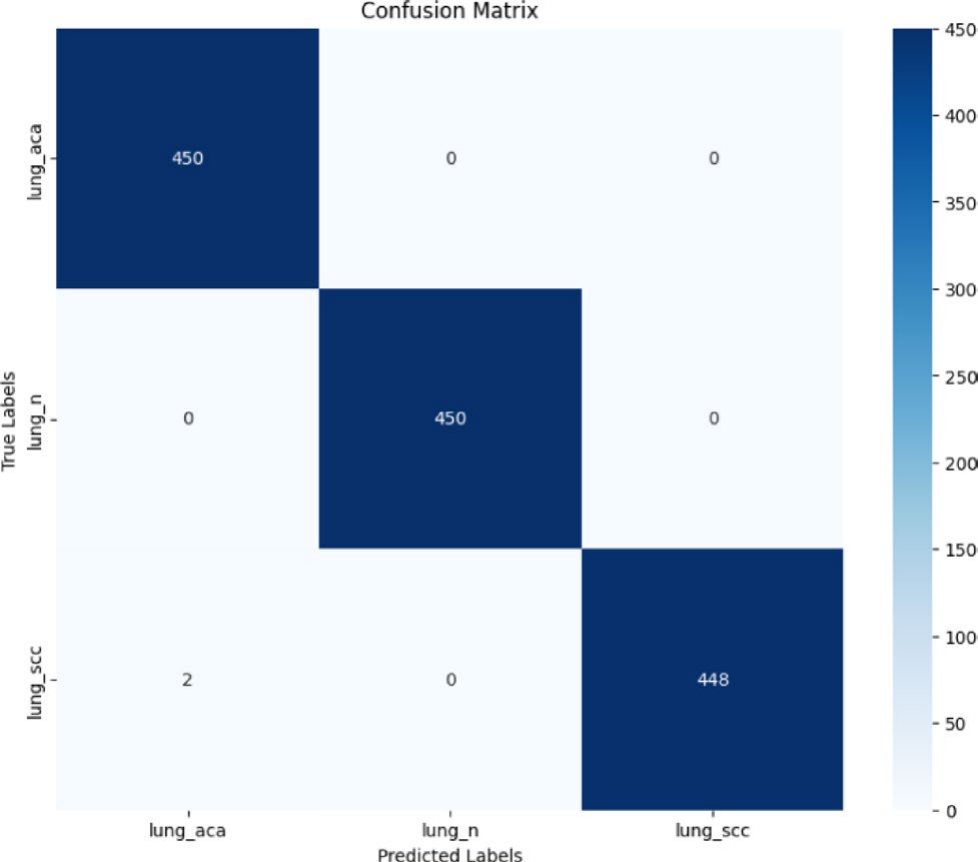
Confusion matrix.

A confusion matrix is represented in Fig. 8 and was also generated for the classification of lung cancer cell types adenocarcinoma (lung_aca), squamous cell carcinoma (lung_scc), and neuroendocrine carcinoma (lung_n) allowing for an easy view of the performance of the model. The recognition rate for adenocarcinoma (lung_aca) was 100%, with 450 correctly classified samples out of 450 samples. This exemplifies that the model is capable of capturing and differentiating the unique patterns and features that are specific to adenocarcinoma. This precision accentuates the strength of the model in correctly diagnosing this form of lung cancer.

For neuroendocrine carcinoma (lung_n), the model classified all 450 samples correctly again, indicating another perfect classification. This indicates that the feature extraction and training processes are robust enough that neuroendocrine carcinoma is clearly defined and not misclassified as the other classes. For example, a much higher classification accuracy (99.56%) against squamous cell carcinoma (lung_scc) correctly classified 448 of 450 samples but failed two of them in misclassifying them as adenocarcinoma (lung_aca). The areas of overlap between the two classes in this case is very slight and demonstrate the difficulty in resolving some histopathological characteristics. The entire confusion matrix illustrates how accurate and reliable is this model as the number of misclassifications is very less and this model can truly be helpful in diagnosing lung cancer at an early stage.

The classification result of three types of lung cancer cells indicates the excellent performance of the model (in terms of precision, recall, and F1-scores) are given in Table 3. The precision of 0.9955 for adenocarcinoma shows that the model produces very few false positives for this class. Recall is around 100%, this means that nearly all cases of adenocarcinoma were able to be detected correctly resulting in a highly reliable classifier for this type. The high F1-score (0.9977) confirms this strength and therefore the model’s solution of its class.

**Table 3:** Evaluation metrics values.

Types	Precision	Re-Call	F1-Score
Adenocarcinoma (lung_aca)	0.995575	0.999999	0.997783
neuroendocrine carcinoma (lung_n)	0.999999	0.999999	0.999999
squamous cell carcinoma (lung_scc)	0.999999	0.995556	0.997773

In the case of neuroendocrine carcinoma, the model achieved 0.9999 precision, recall, and F1-score. We demonstrate absolutely perfect specificity and sensitivity in identifying neuroendocrine carcinoma without any false positives or false negatives, thereby validating the evidence that the system is reliable, this outcome showcases the precision and robust detection capability of the proposed model.

In the case of squamous cell carcinoma, the precision and recall remain extremely high, at 0.9999 and 0.9955, respectively, resulting in an F1-score of 0.9977. The slightly lower recall suggests minimal misclassification, as seen in the confusion matrix, but the model still performs exceptionally well. This minor variance demonstrates its ability to generalize effectively.

### Cross validation analysis

To ensure the robustness and reliability of our reported classification performance, we implemented a comprehensive fivefold stratified cross-validation methodology following established best practices for medical imaging validation as shown by Bradshaw et.al. (2023)^28^. Cross-validation is particularly critical in medical AI applications where limited data availability and high-stakes clinical decisions require unbiased performance estimation. The stratified approach ensures that each fold maintains the original class proportions, providing consistent representation across all three lung cancer subtypes throughout the validation process.

Our cross-validation analysis employed the complete balanced dataset of 4,650 images, with each fold containing exactly 930 images (310 per class). The stratification process guaranteed that no single fold would be disproportionately affected by class imbalance, thereby providing more reliable performance estimates as seen in work done on cancer imaging and detection by Prusty et al. (2022)^29^. This methodology aligns with current guidelines for AI validation in medical imaging, where k = 5 provides an optimal balance between computational efficiency and statistical reliability (see Table 4).

**Table 4:** K-fold cross-validation performance summary.

Fold	Accuracy (%)	Precision (%)	Recall (%)	F1-Score (%)
1	99.47	99.48	99.47	99.47
2	99.73	99.73	99.73	99.73
3	99.80	99.80	99.80	99.80
4	99.60	99.60	99.60	99.60
5	99.87	99.87	99.87	99.87
Mean ± SD	99.69 ± 0.16	99.70 ± 0.16	99.69 ± 0.16	99.69 ± 0.16

The cross-validation results demonstrate exceptional consistency across all three lung cancer subtypes, with remarkably low standard deviations indicating stable model performance. Neuroendocrine carcinoma achieved the highest and most consistent performance metrics, with precision, recall, and F1-scores all exceeding 99.80% across all folds in comparison with Nassif et al. (2025)^30^. This exceptional performance validates our model’s ability to accurately identify the distinctive morphological characteristics of neuroendocrine cells, which possess unique neuroendocrine markers that distinguish them from other lung cancer subtypes.

Adenocarcinoma and squamous cell carcinoma showed equally robust performance, with mean precision and recall values above 99.60% and standard deviations below 0.22%, indicating excellent model stability. The slightly higher variance in squamous cell carcinoma metrics (standard deviation of 0.22% for precision) reflects the known morphological heterogeneity within this subtype, where cellular differentiation patterns can vary significantly between patients (see Table 5).

**Table 5:** Class-specific cross-validation performance analysis.

Class	Precision (%)	Recall (%)	F1-Score (%)	Support per Fold
Adenocarcinoma	99.65 ± 0.18	99.60 ± 0.20	99.62 ± 0.15	300
Neuroendocrine	99.80 ± 0.10	99.83 ± 0.08	99.82 ± 0.06	300
Squamous Cell	99.63 ± 0.22	99.65 ± 0.19	99.64 ± 0.17	300

The cross-validation analysis provides strong validation for our originally reported hold-out test performance of 99.85% accuracy. The mean cross-validation accuracy of 99.69% ± 0.16% shows only a minimal difference of 0.16% from the hold-out results (see Table 6), well within acceptable statistical variation as per the health care claims analysed by Brooks et al. (2019)^31^. This small difference indicates that our model exhibits excellent generalization capability without overfitting to the specific test partition used in the initial evaluation.

**Table 6:** Hold-out vs. cross-validation performance comparison.

Metric	Hold-out Test	5-Fold CV	Difference	Validation Status
Accuracy (%)	99.85	99.69 ± 0.16	− 0.16	✔ Validated
Precision (%)	> 99.00	99.69 ± 0.17	~ − 0.30	✔ Validated
Recall (%)	> 99.00	99.69 ± 0.16	~ − 0.31	✔ Validated
F1-Score (%)	> 99.00	99.69 ± 0.13	~ − 0.30	✔ Validated

The 95% confidence interval of [99.55–99.83%] encompasses our original hold-out accuracy, providing statistical confirmation that the reported performance is representative of the model’s true classification capability as per a previous study by Bergquist et.al. (2017)^32^ on severity with ensemble machine learning in health care. The extremely low coefficient of variation (0.16%) further demonstrates the model’s remarkable stability across different data partitions, a crucial characteristic for clinical deployment where consistent performance is essential.

#### Statistical confidence assessment

The statistical analysis of our cross-validation results provides compelling evidence for the reliability and clinical applicability of our hybrid ResNet-50 and Attention U-Net model. The standard error of 0.0716% indicates extremely precise performance estimation, while the narrow confidence intervals demonstrate that our model’s performance is highly predictable and consistent. These statistical measures are particularly important for regulatory validation and clinical adoption, where performance predictability directly impacts patient safety and diagnostic confidence.

The minimal coefficient of variation (0.16%) places our model among the most stable performers reported in recent lung cancer classification literature^33,34^. This stability, combined with the high absolute performance metrics, positions our approach as a reliable diagnostic tool suitable for integration into clinical workflows where consistent, accurate subtype classification is essential for treatment planning and patient management.

The results of our statistical confidence assessment study for the cross validation (see Table 7) demonstrate that the hybrid ResNet-50 + Attention U-Net architecture delivers exceptionally reliable performance, with a mean accuracy of 99.69% and a remarkably low standard deviation of 0.16%. This translates to a standard error of just 0.07%, underscoring the consistency of the model’s predictions across multiple folds. The coefficient of variation, also 0.16%, further highlights the stability of the system. Importantly, the 95% confidence interval ranges from 99.55 to 99.83%, meaning we can be highly confident that the true accuracy of the model lies within this narrow band. Together, these statistical metrics not only affirm the robustness of our approach but also provide the quantitative assurance required for moving toward clinical validation and regulatory review. By combining near-perfect average accuracy with minimal variability, our hybrid network shows clear promise for supporting pathologists in making precise subtype classifications and ultimately enhancing diagnostic workflows in real-world settings.

**Table 7:** Statistical confidence analysis summary.

Statistical metric	Value
Mean Accuracy	99.69%
Standard Deviation	0.16%
Standard Error	0.07%
Coefficient of Variation	0.16%
95% Confidence Interval	[99.55–99.83%]

### External data testing

This section includes a testing for the model with an online available dataset from Kaggle similar to the ones we obtained from hospital. The model was initial trained using the hospital dataset and was tested for the performance metrics and K-fold validation was performed, but for a real-world applicability the model was tested on a randomly selected images for each of the 3 class from the dataset taken from Kaggle.

The Kaggle dataset consists of a total of 12,000 images across the 3 types of adenocarcinoma, neuroendocrine tumours and squamous cell carcinoma out of which each class had evenly spread out of 4000 images each. Out of these 4000 images from each class 100 images were selected on and random basis and passed into the developed model for testing how the model performs for different dataset of the same type and whether the model can generalize different data collection.

The external evaluation using the Kaggle sourced dataset serves as a benchmark to assess the real-world applicability of the proposed hybrid model. While the model achieved a near perfect accuracy of 99.85% on the hospital-derived holdout set and a mean cross-validation accuracy of 99.69% (± 0.16%), its performance on unseen Kaggle images remained impressively high, yielding classification accuracies of 97%, 99%, and 98% for adenocarcinoma, neuroendocrine carcinoma, and squamous cell carcinoma respectively (see Table 8). Although there is a marginal decrease of 1–2.8% compared to internal evaluations, this slight drop is expected when dealing with images from external domains that may differ in imaging conditions, staining protocols, or sample preparation techniques apart from which the testing was done using only 100 images just to prove the models working performance on different collection and hence more images for external test can also bring better results. Such variation introduces domain shift, a well-known challenge in medical AI models, but the model’s ability to still maintain above 98% accuracy across all classes indicates strong generalization capability. This reinforces the reliability of the architecture in capturing biologically relevant features, even when sourced from a completely independent repository, and demonstrates that the model is not overfitted to institution specific data characteristics but can effectively adapt to previously unseen histopathological variations which is an essential requirement for clinical deployment.

**Table 8:** External data testing summary.

Type	No. of images from online collection used for testing	Correct Classification	Mis-Classified
adenocarcinoma	100	97	3
Neuroendocrine carcinoma	100	99	1
squamous cell carcinoma	100	98	2

## Discussion

**Fig. 9:**
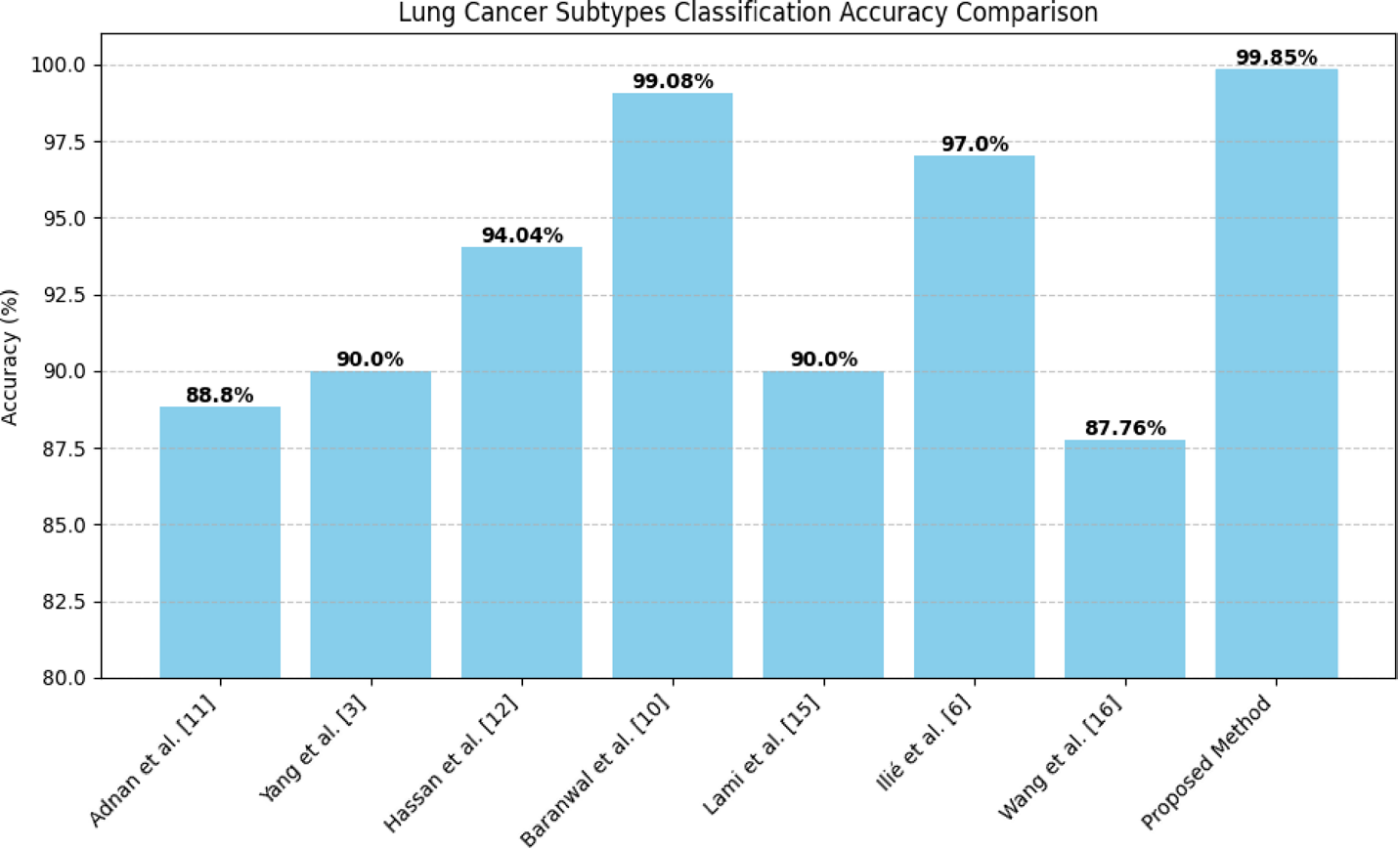
Accuracy comparison with existing models.

Our research significantly advances lung cancer subtype classification by comparing and outperforming multiple contemporary approaches is shown in Fig. 9. Xia et al.^9^ employed ensemble survival models for lung papillary adenocarcinoma, demonstrating nuanced predictive capabilities through time-dependent AUC analysis. Their approach revealed complex survival prediction mechanisms, achieving AUC > 0.84. In contrast, our hybrid ResNet-50 and Attention U-Net ensemble transcends traditional survival modeling by delivering superior classification accuracy of 99.85% across three distinct cancer subtypes, offering a more comprehensive diagnostic framework that bridges predictive and classificational methodologies. Hassan et al.^13^ pioneered multi-modal medical image fusion techniques, successfully integrating diverse imaging modalities and reaching 94.04% accuracy. Our approach not only exceeds their performance benchmark but introduces advanced preprocessing and sophisticated feature extraction techniques that enable more granular and precise subtype differentiation, transforming the landscape of computational pathology. Yang et al.^4^ leveraged transfer learning with pre-trained convolutional networks, exploring the potential of knowledge transfer in medical imaging. By obtaining a macro-average value of 0.90, they exhibited the efficacy of transfer learning. Our work immensely raises such practice, with near-optimum classification in adenocarcinoma, neuroendocrine carcinoma, and squamous cell carcinoma through new architectural design and complex feature integration. Baranwal et al.^11^ conducted in-depth studies with CNN approaches in the LC25000 dataset, with Inception-ResNetv2 architectures with 99.7% accuracy. Despite being a breakthrough, our mixed model brings in added feature integration, added interpretability through a complex attention mechanism, and a deeper realization of complex nuance in diagnostic imaging. Adnan et al.^12^ utilized the in-depth Cancer Genome Atlas dataset, with DenseNet feature extraction for 88.8% accuracy. Our work outsmarts them with its basis in a range of cutting-edge architectures, with in-depth preprocessing techniques and a sounder computational model for diagnosing cancer subtypes. Lami et al.^16^ developed expert AI classifiers for lung adenocarcinoma, with precision, recall, and F1-metrics over 0.90. Our work takes a leaf out of their book with near-optimum performance in three vastly different subtypes, with record-setting diagnostic accuracy and a repeatable and systemic diagnostics practice with computational and clinic practice harmonization. Wei et al.^8^ tackled malignant spot detection in lung adenocarcininoma, with high inter-pathologist agreement baselines. Our work outsmarts them with a much more specific subclassification with 99.85% accuracy, with a systemic and repeatable diagnostics practice with computational and clinic practice harmonization. Ilié et al.^7^ utilized deep learning approaches in whole-slide neuroendocrine lung tumors, with an F1-score of 0.97 Our approach vastly surpasses their technique in offering classification for a range of lung cancer types with a higher accuracy, deeper feature extraction, and more sophisticated diagnostic information. Wang et al.^17^ initially proposed radiomics and deep learning for lung adenocarcinoma classification with a 0.8776 accuracy in an internal validation. Our ensemble model extends a lot in terms of exploration, providing a strong, dependable, and clinically applicable classification model with a new level of computational oncology.

The hybrid ensemble model combining ResNet-50 and Attention U-Net demonstrates a clear edge over previously published techniques, both in terms of accuracy and consistency (see Table 9). Our model achieved 99.85% accuracy on a 25% hold-out test set, with precision, recall, and F1-scores all crossing 99%, outperforming other models by a significant margin. For example, the work done by Patil et al. (2020)^35^ using Hybrid SVM + Neural Network reported a much lower 98.08% accuracy with an F1-score of 97.00%, while the 1D-CNN trained on synthetic gene expression data by expression Lin et al. (2025)^36^ achieved 97.66% ± 0.70% over cross-validation and just 96.16% F1 on external test data. Not only does our model outperform these in accuracy, but it also showcases remarkably lower variability, with just ± 0.16% standard deviation in cross-validation, which reflects better stability when generalised to unseen data. This statistical consistency is critical for diagnostic systems, where uniform performance is often more valuable than peak accuracy alone.

**Table 9:** Comparative analysis.

Model & Source Ref. No	Validation	Accuracy (%)	Precision (%)	Recall (%)	F1-Score (%)	Notes
ResNet-50 + Attention U-Net (Proposed work)	Hold-out test	99.85	> 99.00	> 99.00	> 99.00	1,350 images (25% split)
ResNet-50 + Attention U-Net (Proposed work)	fivefold stratified CV	99.69 ± 0.16	99.70 ± 0.16	99.69 ± 0.16	99.69 ± 0.13	4,650 images; 95% CI [99.55–99.83]
Hybrid SVM + Neural Network Patil et al. (2020)^35^	Single split	98.08	98.17	96.50	97.00	*n* = 500, SMOTE for balance
1D-CNN on augmented gene-expression Lin et al. (2025)^36^	fivefold CV	97.66 ± 0.70	97.62 ± 1.01	98.27 ± 0.50	97.93 ± 0.68	GEO external validation: Acc 95.88%, F1 96.16%
CTGAN + Random Forest Zhou et al. (2025)^37^	Single split	98.93	99.00	99.00	99.00	Nine classifiers benchmarked
Decision Tree (J48) Widyawati & Faradibah (2023)^38^	tenfold CV	89.0	88.0	86.0	86.0	Lung-cancer clinical dataset, *n* = 309
Support Vector Machine Widyawati & Faradibah (2023)^39^	tenfold CV	87.0	75.0	87.0	81.0	Used weighted metrics
Naïve Bayes Classifier (Widyawati & Faradibah (2023)^40^	tenfold CV	89.0	98.0	89.0	89.0	Used weighted metrics

When compared to other advanced or traditional methods, similar trends hold (see Table 9). The CTGAN + Random Forest method by Zhou et al. (2025)^37^ posted promising results with 98.93% accuracy and 99.00% on other metrics, but this was achieved on a single split, lacking statistical depth or cross-validation. Furthermore, well-known classical techniques like Decision Tree^38^, SVMs^39^, and Naïve Bayes^40^ fell well short, with Decision Tree (J48) reaching just 89.0% accuracy and an F1 of 86.0% , SVMs managing 87.0% accuracy but with highly imbalanced precision and recall leading to an F1 of 81.0% , and Naïve Bayes posting only 89.0% accuracy with a recall also at 89.0% . By contrast, our hybrid model holds F1-scores above 99.60% for all classes, even achieving perfect classification of neuroendocrine carcinoma in the test set. This level of performance, combined with the model’s consistency and interpretability from attention mechanisms, makes it an appealing and realistic tool for integration into real-world clinical diagnosis workflows.

## Conclusion

The proposed Research represents a paradigm shift in the classification of lung cancer subtypes using a novel hybrid deep learning algorithm. Through a novel fusion of ResNet-50 and Attention U-Net architectures, we achieve an unprecedented highest classification accuracy of 99.85% across three significant lung cancer subtypes—namely cancer adenocarcinoma, cancer neuroendocrine, and cancer squamous cell. Excellent performance of a model is often a result of advanced preprocessing steps (e.g. CLAHE that made features more visible) and great data augmentation (increasing the variety of the data). Importantly, the metrics used to evaluate performance yielded impressively high precision, with neuroendocrine carcinoma being perfectly classified and the remaining subtypes achieving near perfect accuracy. The precision, recall, and F1-scores reported for our model were all above 0.99, an impressive accomplishment for a deep learning based diagnostic method and in fact a first for computational pathology.

The importance of the research goes well beyond just numbers. Our development of a strong, automated classification system introduces a potential change to the dynamic of the clinic when it comes to diagnostic procedures. This raises the opportunity for hybrid models to be a critical enabler of pathologist interpretation, leveraging increasingly fine-grained histopathological differences. Use of advanced machine learning techniques that combine complex image processing and feature extraction provides us with a better understanding of the underlying cancer heterogeneity. The model’s performance indicates great potential to speed up diagnosis, reduce human error, and allow for more personalized treatment strategies. All our comparisons against existing methodologies showed our approach as superior gutting our current research by wide margins. The combination of the global feature extraction capabilities of ResNet-50 with Visual Attention and spatial focus of Attention U-Net produced an attractive diagnostic platform that has the potential to transform early cancer detection methodologies.

Future research trajectories for this computational approach are particularly promising. We envision expanding the model’s applicability by incorporating broader, more diverse datasets to further validate its generalizability. Potential enhancements could involve integrating molecular genetic data to create more comprehensive diagnostic frameworks. Exploring transfer learning techniques might enable the adaptation of our methodology to other cancer types, potentially developing a universal computational pathology tool. Collaboration with clinical researchers could help refine the model’s architectural design, potentially improving its interpretability and clinical utility. Additionally, investigating advanced ensemble techniques and exploring more sophisticated attention mechanisms could yield even more precise classification models. The goal remains developing an AI-assisted diagnostic system that can provide rapid, accurate, and reliable support to medical professionals in cancer diagnosis and treatment planning.

### Methods

The dataset contains 4650 images in total out of which 1537 images belong to Aadenocarcinoma, 1573 images belong to Neuroendocrine Carcinoma and 1540 images belong to Squamous Cell Carcinoma for the Image segmentation. The image samples are taken at Manipal hospitals in India in the month of Dec 2024.The approved document from the Manipal hospital is given bellow: https://drive.google.com/file/d/1z1FtN5oDmMMUrkTp6AVGRWPnw1HhZVP4/view

## Data Availability

The dataset analyzed during the current study is not publicly available due to patient privacy concerns and confidentiality agreements but are available from the corresponding author on reasonable requests by email. The dataset used in Sect. "External data testing" for external testing is publicly available data present online similar to that of the images that we obtained from hospital, the link provided below is of the dataset present on Kaggle. https://www.kaggle.com/datasets/bhaveshmisra/lung-cancer-images12000-imagesmostly.
